# Establishment and validation of a prognostic nomogram for patients with resectable perihilar cholangiocarcinoma

**DOI:** 10.18632/oncotarget.9104

**Published:** 2016-04-29

**Authors:** Peizhan Chen, Bin Li, Yan Zhu, Wei Chen, Xin Liu, Mian Li, Xiaohua Duan, Bin Yi, Jinghan Wang, Chen Liu, Xiangji Luo, Xiaoguang Li, Jingquan Li, Lijian Liang, Xiaoyu Yin, Hui Wang, Xiaoqing Jiang

**Affiliations:** ^1^ Key Laboratory of Food Safety Research, Institute for Nutritional Sciences, Shanghai Institutes for Biological Sciences, Chinese Academy of Sciences, University of Chinese Academy of Sciences, Shanghai, 200031, P. R. China; ^2^ Biliary Tract Surgery Department I, Eastern Hepatobiliary Surgery Hospital, Secondary Military Medical University, Shanghai, 200433, P. R. China; ^3^ Diagnosis and Treatment Center of Malignant Biliary Tract Diseases, Secondary Military Medical University, Shanghai, 200433, P. R. China; ^4^ Department of Pathology, Changhai Hospital, Secondary Military Medical University, Shanghai, 200433, P. R. China; ^5^ Department of Pancreatobiliary Surgery, The First Affiliated Hospital, Sun Yat-Sen University, Guangzhou, Guangdong, 510080, P. R. China; ^6^ Department of Hepatobiliary Surgery, Navy General Hospital, 100048, P. R. China; ^7^ Key Laboratory of Food Safety Risk Assessment, Ministry of Health, Beijing, 100021, P. R. China; ^8^ School of Life Science and Technology, ShanghaiTech University, Shanghai, 200031, P. R. China

**Keywords:** perihilar cholangiocarcinoma, overall survival, nomogram

## Abstract

As the conventional staging systems have poor prognosis prediction ability for patients with perihilar cholangiocarcinoma (pCCA), we established and validated an effective prognostic nomogram for pCCA patients based on their personal and tumor characteristics. A total of 235 patients who received curative intent resections at the Eastern Hepatobiliary Surgery Hospital from 2000 to 2009 were recruited as the primary training cohort. Age, preoperative CA19-9 levels, portal vein involvement, hepatic artery invasion, lymph node metastases, and surgical treatment outcomes (R0 or R1/2) were independent prognostic factors for pCCA patients in the primary cohort as suggested by the multivariate analyses and these were included in the established nomogram. The calibration curve showed good agreement between overall survival probability of pCCA patients for the nomogram predictions and the actual observations and the concordance index (C-index) was 0.68 (95% CI, 0.61-0.71). The C-index values and time-dependent ROC tests suggested that the nomogram is superior to the conventional staging systems including the Bismuth-Corlette, Gazzaniga, Memorial Sloan Kettering Cancer Center (MSKCC), American Joint Committee on Cancer (AJCC) TNM 7^th^ edition, and Mayo Clinic. The nomogram also performed better than the traditional staging system in the internal cohort with 93 pCCA patients from the same institution and an external validation cohort including 84 pCCA patients from another institution in predicting the overall survival of the pCCA patients as suggested by the C-index values and the time-dependent ROC tests. In summary, the proposed nomogram has superior predictive accuracy of prognosis for resectable pCCA patients.

## INTRODUCTION

Cholangiocarcinoma, which occurs in epithelial cells within the biliary tree, is a relatively rare malignancy, but its incidence has been rising over the past several decades [1]. Cholangiocarcinoma accounts for about 3% of all gastrointestinal cancers worldwide [2]. Concerning the anatomical location, 60-70% of cholangiocarcinomas are perihilar, 20-30% are distal, and about 10% are intrahepatic [3]. According to the National Cancer Institute's SEER program, the clinical outcomes for patients with perihilar cholangiocarcinoma (pCCA) are poor, with the 5-year survival rate ranging from 25% to 35% for localized cancer to less than 2% for patients with distant metastases [4]. Cholangiocarcinoma is characterized by a poor response to chemotherapy and radiation treatments, and the benefits to these treatments are not yet fully understood [5]. Surgical treatment, especially resections and liver transplantations, are most effective; however, only a few patients are eligible for such treatments because of late diagnoses and the complexity of the disease.

Identification of patients who may have poorer prognoses is required for the extensive treatments to improve their outcomes. For pCCAs, there are several staging systems, including the Bismuth-Corlette, Gazzaniga, and MSKCC (Memorial Sloan-Kettering Cancer Center) staging systems, which are widely used to predict the resectability of the tumor, but their roles in prognostic prediction are limited [6–8]. Further, the American Joint Committee on Cancer (AJCC) TNM and the Mayo Clinic systems have been proposed to predict the outcomes for the patients; however, they consider only a limited number of factors related to prognosis and lack accuracy [6, 9].

Nomograms provide a simple, graphics-based prognostic system to predict, for individual patients, their long-term survival based on personal and clinical characteristics. They have been proved to be more accurate than conventional systems for a variety of malignant neoplasms, including hepatocellular carcinoma, pancreatic adenocarcinoma, and intrahepatic cholangiocarcinoma [10–12]. For pCCAs, there is a lack of systematic evaluation of prognostic factors, especially for patients who have received curative intent surgery treatments. Here, clinical factors that may influence the prognosis of pCCA patients were systematically evaluated, and a nomogram was established to predict the long-term outcomes for pCCA patients after surgical treatment.

## RESULTS

### Participants characteristics of the primary training cohort

A total of 235 eligible patients were recruited from the Eastern Hepatobiliary Surgery Hospital of the Second Military Medical University as the primary training cohort between the years 2000 to 2009, and they were recognized as the primary training cohort to establish the prognostic nomogram. The follow-up time ranged from 1.7 to 143.9 months, with a median time of 23.3 months. The median overall survival (OS) time of the patients was 24.2 months (range, 1.7 to 75.0 months). The cumulative 1-, 3-, and 5-year survival rates of the patients were 77.4%, 28.9%, and 11.1%, respectively. 177 (75.3%) patients received R0 (complete) resections, and 58 (24.7%) received R1 (microscopic residual tumor) or R2 (macroscopic residual tumor) resections. The 1-, 3- and 5- year survival rates for patients with R0 and R1/R2 treatment were 84.8%, 35.0%, 24.3%, and 60.3%, 15.5%, 6.9%, respectively. The basic characteristics for the patients are shown in Table 1.

**Table 1:** Basic characteristics for the recruited participants in the primary training cohort (N = 235), internal validation cohort (N = 93), and external validation cohort (N = 84)

Demographic or characteristic factor	Training cohort (N = 235)	Internal cohort (N = 93)	P-value^*^	External cohort (N = 84)	P-value^#^
Age, years (± SD)	56.8 ± 11.2	58.7 ± 9.1	0.119	58.4 ± 11.9	0.298
Sex (Male/Female)	158/77 (67.2%/32.8%)	54/39 (58.1%/41.9%)	0.118	49/35 (58.3%/41.7%)	0.142
Tumor size, cm (± SD)	2.75 ± 1.04	2.61 ± 1.17	0.323	3.09 ± 1.28	0.028
Bismuth staging (I/II/IIIa/IIIb)	17/52/56/110 (7.2%/22.1%/23.8%/46.8%)	13/21/24/35 (14.0%/22.6%/25.8%/37.6%)	0.284	2/13/32/37 (2.4%/15.5%/38.1%/44.0%)	0.037
Gazzaniga staging (I/II/III/IV)	60/133/34/8 (25.5%/56.6%/14.5%/3.4%)	18/55/20/0 (19.4%/59.1%/21.5%/0.0%)	0.097	27/10/4/4 (60.0%/22.2%/8.9%/8.9%)	< 0.001
MSKCC T staging (T1/T2/T3)	179/25/31 (76.2%/10.6%/13.2%)	75/7/11 (80.6%/7.5%/11.8%)	0.628	29/10/6 (64.4%/22.2%/13.3%)	0.093
AJCC T staging (T1/T2/T3/T4)	16/143/28/48 (6.8%/60.9%/11.9%/20.4%)	2/61/15/15 (2.2%/65.6%/16.1%/16.1%)	0.220	0/27/10/8 (0.0%/60.6%/22.2%/17.8%)	0.104
AJCC N staging (N0/N1-2)	172/63 (73.2%/26.8%)	67/26 (72.0%/28.0%)	0.833	25/20 (55.6%/44.4%)	0.018
Differentiation (Low/Medium/High)	26/199/10 (11.1%/84.7%/4.3%)	1/90/2 (1.1%/96.8%/2.2%)	0.007	7/33/5 (15.6%/73.3%/11.1%)	0.103
Portal vein invasion					
None	178 (75.7%)	75 (80.6%)		56 (66.7%)	
Ipsilateral portal vein branch	25 (10.6%)	7 (7.5%)		9 (10.7%)	
Bifurcation	10 (4.3%)	2 (2.2%)		8 (9.5%)	
Bifurcation plus portal vein branch	10 (4.3%)	2 (2.2%)		5 (6.0%)	
Main portal vein encasement	13 (5.5%)	7 (7.5%)	0.571	6 (7.1%)	0.355
Hepatic artery invasion					
None	203 (86.4%)	70 (75.3%)		62 (73.8%)	
Hepatic artery branch	26 (11.1%)	19 (20.4%)		22 (26.2%)	
Main hepatic artery	6 (2.6%)	4 (4.3%)	0.052	0 (0.0%)	0.002
Perineural invasion (No/Yes)	116/119 (49.4%/50.6%)	19/74 (20.4%/79.6%)	< 0.001	49/35 (58.3%/41.7%)	0.158
Liver invasion (No/Yes)	208/27 (88.5%/11.5%)	70/23 (75.3%/24.7%)	0.003	67/17 (79.8%/20.2%)	0.046
Spigelian lobe resection (No/Yes)	128/107 (54.5%/45.5%)	53/40 (57.0%/43.0%)	0.679	50/34 (59.5%/40.5%)	0.423
Radiotherapy (No/Yes)	185/50 (78.7%/21.3%)	82/11 (88.2%/11.8%)	0.047	81/3 (96.4%/3.6%)	< 0.001
Chemotherapy (No/Yes)	187/48 (79.6%/20.4%)	78/15 (83.9%/16.1%)	0.373	76/8 (90.4%/9.6%)	0.024
Surgery treatment (R0/R1 or 2)	177/58 (75.3%/24.7%)	68/25 (73.1%/26.9%)	0.679	59/25 (70.2%/29.8%)	0.362
ECOG status (0/1/2-3)	19/138/78 (8.1%/58.7%/33.2%)	3/62/28 (3.2%/66.7%/30.1%)	0.199	21/47/16 (25.0%/56.0%/19.0%)	< 0.001
CA19-9 level (≤73.5/73.6-325.0/≥325.1 U/ml)	78/78/79 (33.2%/33.2%/33.6%)	24/42/27 (25.8%/45.2%/29.0%)	0.122	28/24/32 (33.3%/28.6%/38.1%)	0.680
Mayo Clinic (I/II/III-IV)	12/107/116 (5.1%/45.5%/49.4%)	0/46/47 (0%/49.5%/50.5%)	0.083	6/19/59 (7.1%/22.6%/70.2%)	0.001
Vascular encasement (No/Yes)	167/68 (71.1%/28.9%)	63/30 (67.7%/32.3%)	0.554	46/38 (54.8%/45.2%)	0.007
AJCC TNM staging (I/II/III/IV)	14/113/54/54 (6.0%/48.1%/23.0%/23.0%)	1/46/27/19 (1.1%/49.5%/29.0%/20.4%)	0.197	5/23/33/23 (6.0%/27.4%/39.3%/27.4%)	0.005

* P-value for the comparison between training cohort and internal validation cohort.

# P-value for the comparison between training cohort and external validation cohort.

**Abbreviations:** AJCC, American Joint Committee on Cancer; ECOG, Eastern Cooperative Oncology Group; MSKCC, Memorial Sloan Kettering Cancer Center; SD, Standard Deviation.

### Overall survival prognostic factors for pCCA patients

The univariate analyses suggested that age, vascular encasement of the tumor, Gazzaniga stage, MSKCC stage, TNM stage, portal vein invasion status, hepatic artery invasion status, Eastern Cooperative Oncology Group (ECOG) status, plasma CA19-9 levels, surgery treatment outcomes (surgical margin), lymph node metastases, and Mayo Clinic stage were significantly associated with OS (Table 2) in the primary training cohort. Non-linear effects were evident for the continuous variant, age, on the hazard ratio for the OS of the participants (Supplementary Figure S1), and a restricted cubic spline model with three knots for age was applied in further analyses based on the optimized tests. With backward stepwise selection methods, age, treatment outcomes, portal vein invasion status, hepatic artery invasion, preoperative CA19-9 levels, and lymph node metastases were found to be independent prognosis factors for pCCA patients (Table 3).

**Table 2:** Univariate analysis for the associations between the personal and clinical characteristics and the OS for pCCA patients in the primary training cohort (N = 235)

Demographic or characteristic factor	HR (95% CI)	P-value
Age (per year)	1.02 (1.01-1.03)	0.016
Sex (Female vs. Male)	0.99 (0.73-1.34)	0.940
Tumor size (per cm)	1.08 (0.95-1.24)	0.233
Differentiation		
Medium vs. Low	0.70 (0.45-1.07)	0.101
High vs. Low	0.44 (0.19-1.03)	0.060
Bismuth staging		
II vs. I	0.90 (0.51-1.61)	0.730
IIIa vs. I	1.11 (0.93-1.97)	0.712
IIIb vs. I	1.28 (0.75-2.18)	0.368
Gazzaniga staging		
II vs. I	1.30 (0.92-1.83)	0.138
III vs. I	2.71 (1.70-4.32)	< 0.001
IV vs. I	2.40 (1.13-5.11)	0.023
MSKCC T staging		
T2 vs. T1	1.03 (0.65-1.65)	0.889
T3 vs.T1	2.11 (1.40-3.18)	< 0.001
AJCC T staging		
T2 vs. T1	1.18 (0.67-2.05)	0.568
T3 vs. T1	1.27 (0.65-2.48)	0.491
T4 vs. T1	2.36 (1.28-4.35)	0.006
AJCC N staging (N1/2 vs. N0)	1.61 (1.17-2.22)	0.004
Portal vein involvement		
Ipsilateral portal vein branch vs. None	1.04 (0.65-1.66)	0.865
Bifurcation vs. None	1.20 (0.59-2.46)	0.610
Bifurcation plus portal vein branch vs. None	1.68 (0.82-3.44)	0.156
With main portal vein encasement vs. None	7.49 (4.02-13.95)	< 0.001
Hepatic artery invasion		
Branch vs. None	1.78 (1.15-2.74)	0.009
Main hepatic artery vs. None	4.63 (2.03-10.56)	< 0.001
Perineural invasion (Yes vs. No)	1.09 (0.82-1.44)	0.571
Liver invasion (Yes vs. No)	0.85 (0.55-1.33)	0.477
Spigelian lobe resection (Yes vs. No)	0.91 (0.68-1.21)	0.498
Radiotherapy (Yes vs. No)	1.01 (0.71-1.43)	0.966
Chemotherapy (Yes vs. No)	1.08 (0.77-1.52)	0.662
Surgery treatment outcome		
R1/2 vs. R0	1.94 (1.41-2.66)	< 0.001
ECOG status		
I vs. 0	2.02 (1.09-3.76)	0.026
II-III vs. 0	2.08 (1.09-3.95)	0.026
CA19-9 level (U/ml)		
Tertile 2 (73.5-325.0) vs. Tertile 1 (≤ 73.5)	1.63 (1.14-2.32)	0.008
Tertile 3 (≥ 325.1) vs. Tertile 1 (≤ 73.5)	2.39 (1.68-3.41)	< 0.001
Vascular encasement		
Yes vs. No	1.65 (1.21-2.25)	0.001
AJCC TNM staging		
II vs. I	1.18 (0.65-2.16)	0.586
III vs. I	1.63 (0.86-3.09)	0.134
IV vs. I	2.44 (1.28-4.63)	0.007
Mayo Clinic		
II vs. I	1.81 (0.88-3.73)	0.109
III-IV vs. I	2.31 (1.12-4.77)	0.023

**Abbreviations:** 95% CI, 95% confidential interval; AJCC, American Joint Committee on Cancer; ECOG, Eastern Cooperative Oncology Group; HR, hazard ratio; MSKCC, Memorial Sloan Kettering Cancer Center.

**Table 3:** Multivariate analysis for the primary training cohort (N = 235). The continuous variant, age, was transformed with the restricted cubic spline model with 3 knots

Demographic or characteristic factor	HR (95% CI)	P-value
Age (≤ 57.5 years, per year)	1.06 (1.02-1.09)	0.001
Age' (> 57.5 years, per year)	0.94 (0.91-0.98)	0.002
Surgery treatment outcome (R1/2 vs. R0)	1.52 (1.09-2.12)	0.014
Portal vein involvement		
Main vs. Branch or None	5.51 (2.88-10.55)	< 0.001
Hepatic artery invasion		
Branch vs. None	1.58 (1.01-2.47)	0.048
Main hepatic artery vs. None	3.75 (1.57-8.99)	0.003
AJCC N staging (N1/2 vs. N0)	1.53 (1.10-2.12)	0.012
CA19-9 (U/ml)		
Tertile 2 (73.6-325.0) vs. Tertile 1 (≤ 73.5)	1.70 (1.18-2.44)	0.004
Tertile 3 (≥ 325.1) vs. Tertile 1 (≤ 73.5)	2.30 (1.60-3.30)	< 0.001

**Abbreviations:** 95% CI, 95% confidential interval; AJCC, American Joint Committee on Cancer; HR, hazard ratio.

### Prognostic nomogram of overall survival for pCCA patients

**Figure 1:**
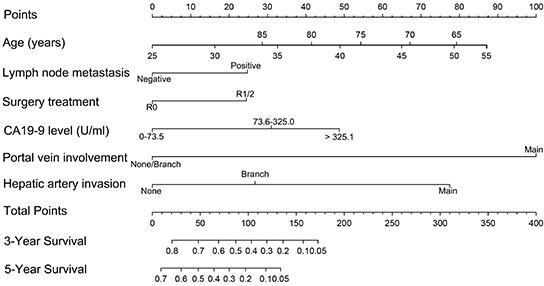
The survival nomogram for the pCCA patients (To use the nomogram, the individual patient's value is located on each variable axis, and a line is drawn upward to determine the risk score for each variant. The sum of these scores is located on the total points axis, and a line is drawn downward to the survival axes to determine the probability of 3- or 5-year survival).

**Figure 2:**
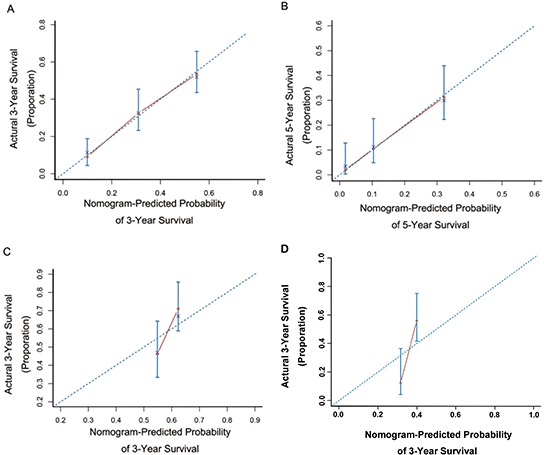
Calibration curves for predicting patient survival at **A.** 3 years and **B.** 5 years in the primary cohort and at 3 years in the internal validation cohort **C.** or external validation cohort **D.** Nomogram-predicted probability of OS is plotted on the *x*-axis; actual OS is plotted on the *y*-axis.

Based on the independent prognostic factors, a nomogram to predict the OS of the pCCA patients was established (Figure 1). The C-index for the OS prediction was 0.68 (95% CI, 0.61 to 0.71). The calibration plot for the probability of survival at 3 or 5 years after surgery showed an optimal calibration between the deaths from the prediction of the nomogram and those derived from the cumulative incidence estimates (Figure 2A and 2B).

### Comparison of predictive accuracy for overall survival between the nomogram and the conventional staging systems

**Figure 3:**
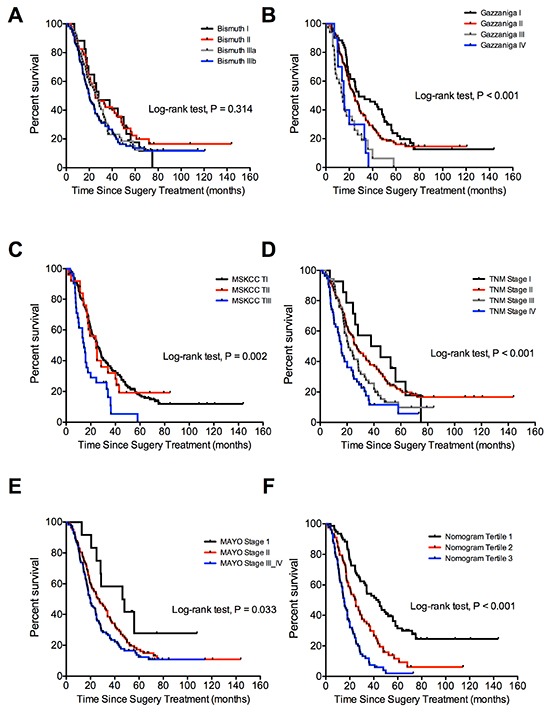
Comparison of the staging systems in the prediction of the OS for pCCA patients in primary training cohort (**A.** Bismuth-Corlette; **B.** Gazzaniga; **C.** MSKCC; **D.** AJCC TNM (seventh edition); **E.** Mayo Clinic; **F.** Nomogram risk score).

The predictive accuracy for the derived nomogram and the conventional staging systems for pCCA patients were compared with the C-index criteria and time-dependent ROC curves. The TNM and Mayo Clinic staging systems showed better prognostic stratification for pCCA patients than the Bismuth, Gazzainiga, and MSKCC systems (Figure 3). In the primary cohort, the nomogram displayed better accuracy in predicting both short- and long-term survival, as determined by time-dependent ROC curves. The C-index of the nomogram (0.68) was significantly higher than the conventional TNM (0.58), Mayo Clinic (0.56), Bismuth-Corlette (0.55), Gazzaniga (0.58), and MSKCC (0.55) staging systems (P < 0.05). The time-dependent ROC curves also suggested that the predictive accuracy for the nomogram was better than the conventional staging systems (P < 0.05) at any specific time (Supplementary Figure S2).

### Internal validation of the prognostic prediction for nomogram

The internal validation cohort comprised 93 patients recruited from the same institution as the primary training cohort between the years 2010 to 2011. The detailed information for these patients is summarized in Table 1. The median follow-up time was 40.0 months (range, 1.9 to 52.0 months) and the median OS was 45.0 months (range, 1.9 to 52.0 months). The rates for deaths at 1 and 3 years post-surgery were 12.9% and 38.7%, respectively. The C-index of the nomogram for predicting the OS was 0.65 (95% CI, 0.56 to 0.74), and the calibration curve showed an optimal calibration between the nomogram prediction and the observation for the probability of 3-year survival (Figure 2C). For the validation cohort, the C-index for the nomogram (0.65) was significantly higher than that for the conventional staging systems. The C-index was 0.61 for the TNM, 0.56 for the Mayo Clinic, 0.54 for the Bismuth, 0.55 for the Gazzaniga, and 0.54 for the MSKCC staging systems. The predictive accuracy for the nomogram was higher than that for any of the conventional staging systems, as indicated by the time-dependent ROC curves (Supplementary Figure S3). The Kaplan-Meier survival plots for the conventional staging systems and the predictive results of the nomogram in the validation cohort are shown in Supplementary Figure S4.

### External validation of the prognostic prediction for the nomogram

84 patients recruited from the First Affiliated Hospital of Sun Yat-Sen University between the years 2005 and 2011 were recognized as the external validation cohort. Detailed information is provided in Table 1. The follow-up time ranged from 3.0 to 117.1 months, with a median of 20.0 months. The median OS of the patients was 24.0 months (range, 3.0 to 117.1 months). The death rate was 25.0% and 56.0% for the patients at 1- and 3-years, respectively. The C-index of the nomogram in OS predicting was 0.68 (95% CI, 0.61 to 0.75), and the calibration curve showed an optimal calibration between the nomogram prediction and the observation of the survival probability at 3-year after the surgery (Figure 2D). The C-index for the nomogram was higher than those for the conventional staging systems. The C-index was 0.63 for Mayo Clinic, 0.59 for TNM, 0.58 for Bismuth, 0.52 for Gazzaniga, and 0.51 for MSKCC staging systems. The predictive accuracy for the nomogram was higher than conventional staging systems, as indicated by the time-dependent ROC curves (Supplementary Figure S5). The Kaplan-Meier survival plots for the conventional staging systems and the nomogram in the external validation cohort are in Supplementary Figure S6, which shows that the nomogram is more accurate in distinguishing those patients with worse from those with better OS (log-rank test, P < 0.001 for tertile categorized nomogram, and P = 0.008 to 0.571 for conventional systems).

## DISCUSSION

Effective prediction of the short- and long-term survival of patients can guide clinical treatments. For pCCA, the Bismuth-Corlette, Gazzaniga, and MSKCC staging systems are most widely used to assess the resectability of tumors [6]. These systems were created based on the location and extent of cancer in the biliary tree, the portal vein invasion of the tumor, and lobar atrophy status; however, their efficacies as prognostics are limited [6]. The AJCC TNM staging was based only on pathological information of the resectable tumors and was expected to be useful in prediction for the OS of pCCA patients undergoing surgical resection [13, 14]; however, it has a lack of accuracy. Recently, the Mayo Clinic staging system showed an optimal stratification for pCCA patients receiving liver transplants or best supportive care [9]; however, the concordance score for patients who received resections was poor. This staging system encompasses only preoperative prognostic factors: ECOG status, CA19-9 levels, vascular encasement of the tumor, tumor number, tumor size, and metastasis [9]. The pathological characteristics of tumors, which influence the prognosis of the pCCA patients, are not included.

Here, we proposed a nomogram to predict the OS for those patients who received resections; the nomogram showed better accuracy than the conventional TNM and Mayo Clinic staging systems. We found that age, CA19-9 level, TNM stage, portal vein invasion, and the treatment outcomes (R0 and R1) were independent prognostic factors for pCCA patients. CA19-9 has been widely used as a diagnostic or prognostic marker for various types of cancers, including pCCA, colorectal cancer, and gastric cancer [15–17]. pCCA patients with preoperative CA19-9 levels < 150 U/ml showed better OS than those with higher CA19-9 levels [18]. pCCA patients with CA19-9 levels > 100 U/ml were associated with increased risk of recurrence for patients who received liver transplants [19], and pCCA patients with blood levels of CA19-9 over 1,000 U/ml were independently associated with worse OS [9]. We also found a negative association between the preoperative plasma CA19-9 levels and the OS of pCCA patients; however, the underlying mechanisms for the aberrant blood CA19-9 levels for pCCA patients are unknown. Lymph node metastasis was an independently adverse factor pCCA patients, an observation validated in other clinical studies [20, 21]. Several previous studies have found that vascular encasement, ECOG status, and the AJCC T stage are associated with pCCA prognosis [9, 22]. The prognostic effects of these factors are consistent with our current study; however, the multivariate analyses suggest that they were not independent prognostic factors for pCCA patients; this may be due to the high correlation with other variants, including portal vein invasion, hepatic artery invasion, and lymph node metastases (data not shown).

The prognostic effects of perineural invasion for patients with extrahepatic cholangiocarcinoma have been determined, showing that patients with perinerual invasion had worse prognoses relative to those who were negative in univariate but not multivariate analyses [23]. In our study, perineural invasion was not associated with the prognosis for pCCA patients, which may be due to the fact that extensive autonomic nerve dissection was performed for those patients. Complete surgical removal of the tumor is the standard treatment for resectable pCCAs, and the histological ductal margin status (R0 or R1/2) is associated with the OS of pCCA patients [24, 25]. We found that patients who received R0 treatment showed better OS than those with R1/2 treatment, which suggested that curative surgery is necessary to achieve a better prognosis. Portal vein invasion may affect the resectability of the pCCA patients and further influence the prognosis of the patients. Here, we found that bifurcation or ipsilateral branch portal vein involvement was not significantly associated with treatment outcomes (R0 or R1/2, data not shown) or with OS [26]. However, invasion of the main portal vein was independently associated with treatment outcomes (R0 or R1/2, data not shown) and with worse OS compared to those without main portal vein invasion. Although hepatic artery invasion had no significant effect on the resectability of pCCA tumors (data not shown), it was significantly associated with a worse prognosis of the patients. Patients with invasion of the branch hepatic artery or main hepatic artery showed a worse OS compared to those without hepatic artery invasion, because hepatic artery invasion may promote the metastasis of pCCA tumors.

Whether postoperative treatments, including radiation or chemotherapy, will benefit pCCA patients is controversial [18]. In our primary study cohort, 50 (21.3%) patients and 48 (20.4%) patients received radiation or chemotherapy treatment, respectively, after surgery. No significantly improved OS of these patients was evident regardless of the R0 or R1/2 resection, consistent with results of previous studies [18, 27]. However, other studies have shown a benefit of OS for those patients with positive resection margins (R1/2) or curative resected patients (R0) who received radiation treatment [5, 28]. More studies are warranted to address the optimal radiation dosing and postoperative treatments to improve the outcomes for pCCA patients.

Recently, Koerkamp et al. proposed a predictive nomogram for pCCA patients [29]. Three prognostic factors, differentiation, lymph node metastasis, and the resection margin status, were independently associated with disease-specific survival of pCCA patients after resections [29]. However, the sample size in the primary cohort was relatively small compared to that in the current study, and the predictive accuracy was not compared between the different staging systems, except for the TNM staging system. Due to a limited number of samples, the investigators did not determine the prognostic effects for portal vein or hepatic artery invasion on the DSS of the patients [29]. The prognostic effects for pre-operative CA19-9 levels were also not determined due to missing values [29]. Koerkamp et al. found that tumor differentiation was independently associated with the disease-specific survival of the pCCA patients [29]; however, there was a marginally significant association between tumor differentiation and the OS of pCCA patients in the primary cohort of the current study. Tumor differentiation was not included in our nomogram according to the stepwise multiple variants regression analyses. Whether tumor differentiation is independently associated with the prognosis of the pCCA patients needs to be elucidated with more investigations.

We acknowledge that there are several limitations for the current study. First, the sample size was relatively small in the internal and external cohorts due to the low incidence of pCCA. Second, the nomogram created here was applicable only to those patients who were eligible for resections. Whether the nomogram would be applicable to patients who were not eligible for curative intent surgery is not known. Third, although the concordance C-index for the nomogram is significantly higher than those for the AJCC TNM and the Mayo Clinic staging systems, the C-index value is still suboptimal (less than 0.70), which suggests that there are other prognostic factors that influence the OS of pCCA patients. More studies are warranted to discover factors that affect the OS for pCCA patients after surgery.

In conclusion, we have developed a reliable nomogram to predict the OS of pCCA patients who receive resections. Although the nomogram is superior to the current staging systems, more studies are required to determine if it is applicable to other groups of patients.

## MATERIALS AND METHODS

### Study design and patient recruitment

A retrospective study was performed for a cohort of pCCA patients who received curative intent surgery from 2000 to 2009 at the Eastern Hepatobiliary Surgery Hospital of the Second Military Medical University. Included were patients with non-metastatic pCCA, did not receive previous anticancer treatments, and had no history of other malignancies. Excluded were patients with intrahepatic or distal cholangiocarcinoma, tumors of uncertain origin or probable metastatic liver tumors, metastatic disease, a mixed type of primary liver cancer with a tumor thrombus perihilar to the bile duct, or non-resectable tumors (Bismuth type IV or Bismuth IIIa or IIIb with contralateral portal vein invasion). Also excluded were patients who died within one month after surgery, which may be caused by postoperative complications. 235 eligible patients were included in the primary training cohort study.

From January 2010 to December 2011, an internal independent validation cohort of 93 pCCA patients who received surgery with curative intent in the same hospital were prospectively recruited with same inclusion and exclusion criteria as the primary cohort.

From 2005 to 2011, 84 pCCA patients that received the curative intent surgery treatment in the First Affiliated Hospital of Sun Yat-Sen University were retrospectively recruited in the current study with the same inclusion and exclusion criteria as the primary training cohort. The last follow-up was performed in September 2015. These patients were recognized as the external validation cohort.

Each participant provided written consent, and the institutional review boards of the Second Military Medical University and the Ethnic Committee of the First Affiliated Hospital of Sun Yat-Sen University have approved the study.

### Data collection

For each participant, baseline personal information was obtained from the admission records. The clinicopathologic characteristics were retrieved from the medical records and assessed by clinicians based on laboratory tests and image evaluations. The treatment methods and related outcomes were retrieved from medical records. The outcomes were followed from January 2001 to June 2014 at half-year intervals in the training cohort and internal cohort. The follow-up was performed from January 2008 to September 2015 with half-year intervals in the external validation cohort. Follow-up information, including metastasis, recurrence, or death, was collected by checking the medical records or by telephone calls.

### Categorization of patients in different staging systems

For each patient, the disease stage was defined according to the criteria of the five staging systems (the seventh edition of the AJCC TNM classification 7^th^ edition, Bismuth-Corlette, MSKCC, Gazzaniga, and Mayo Clinic) [6–9].

### Statistical analyses

Overall survival (OS), defined as the time from the day of surgery to any cause of deaths, was considered as the primary outcome. Associations between the personal and clinical characteristics and the OS were evaluated with Kaplan-Meier plots and further assessed with log-rank tests. Multivariate analyses with the Cox proportional hazards model were performed to identify independent predictors of OS. In the primary cohort, the distribution of CA19-9 levels was categorized into three equal groups due to the upper limit of the test methods. For inclusion in the final model, effects of the continuous variant, age, was explored using restricted cubic splines, as a non-linear relationship was evident between age and the relative hazard risk.

Based on results of the multivariate analyses, the nomogram was created, and the final model was selected by the backward step-down method with the Akaike information criterion. The performance of the nomogram was evaluated with the Harrell concordance index (C-index) and internally validated with 1000 bootstrap samples and calibration plots [30]. To compare the prediction capacity of the staging system and the nomogram, we calculated the C-index value for each staging system and assessed the accuracy of the staging systems and the nomogram by time-dependent receiver operating characteristic (ROC) curves [31]. All statistical analyses were two-sided and conducted in with R software (www.r-project.org) and related packages. Statistical significance was defined as P < 0.05.

## SUPPLEMENTARY FIGURES



## Abbreviations

pCCA: perihilar cholangiocarcinoma
MSCKK: Memorial Sloan Kettering Cancer Center
AJCC: American Joint Committee on Cancer
ROC: receiver operating characteristic
C-inex: concordance index
DSS: disease-specific survival
ECOG: Eastern Cooperative Oncology Group
SD: Standard Deviation
