# Supplementary material for: Establishment and validation of a prognostic nomogram for patients with resectable perihilar cholangiocarcinoma
Source: Oncotarget. 2016 Apr 29;7(24):37319–30. doi: 10.18632/oncotarget.9104 (PMC5095079; doi:10.18632/oncotarget.9104)
Supplement: Supplementary file 1 [file oncotarget-07-37319-s001.pdf]

SUPPLEMENTARY FIGURES

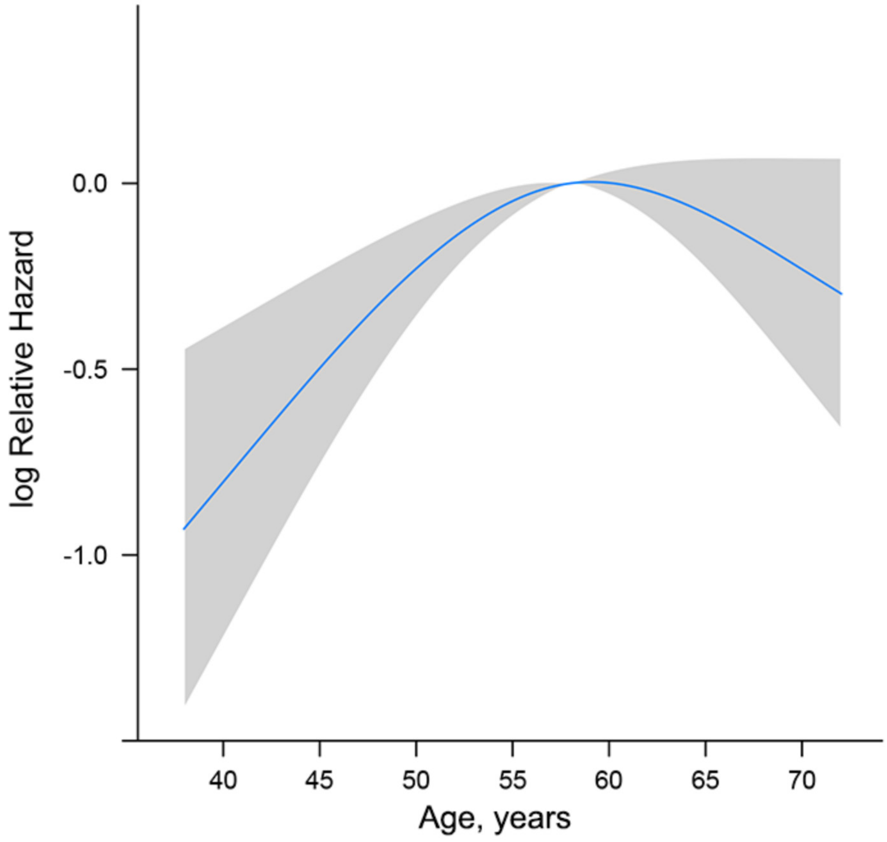

Supplementary Figure S1: Restricted cubic splines transformation of the continuous variant, age, in univariate analyses.

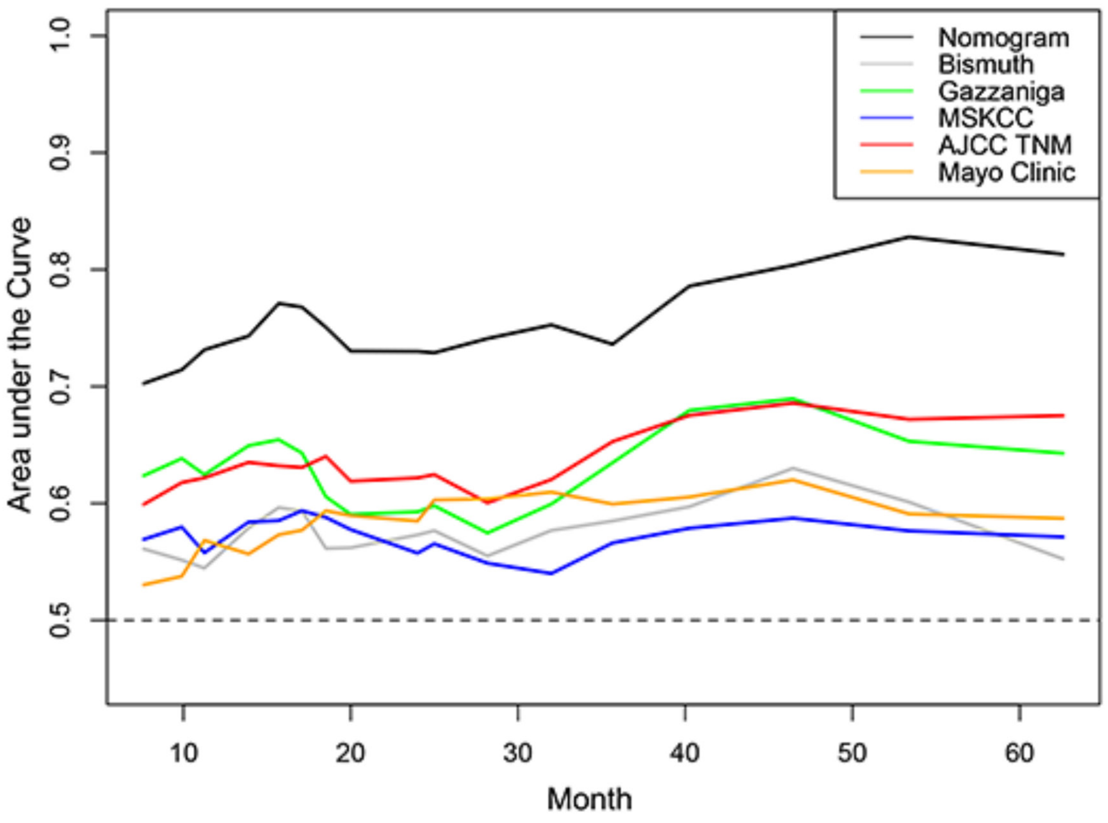

Supplementary Figure S2: Comparison of the time-dependent areas under the ROC curves of prediction by the nomogram and by the conventional staging systems in the primary cohort.

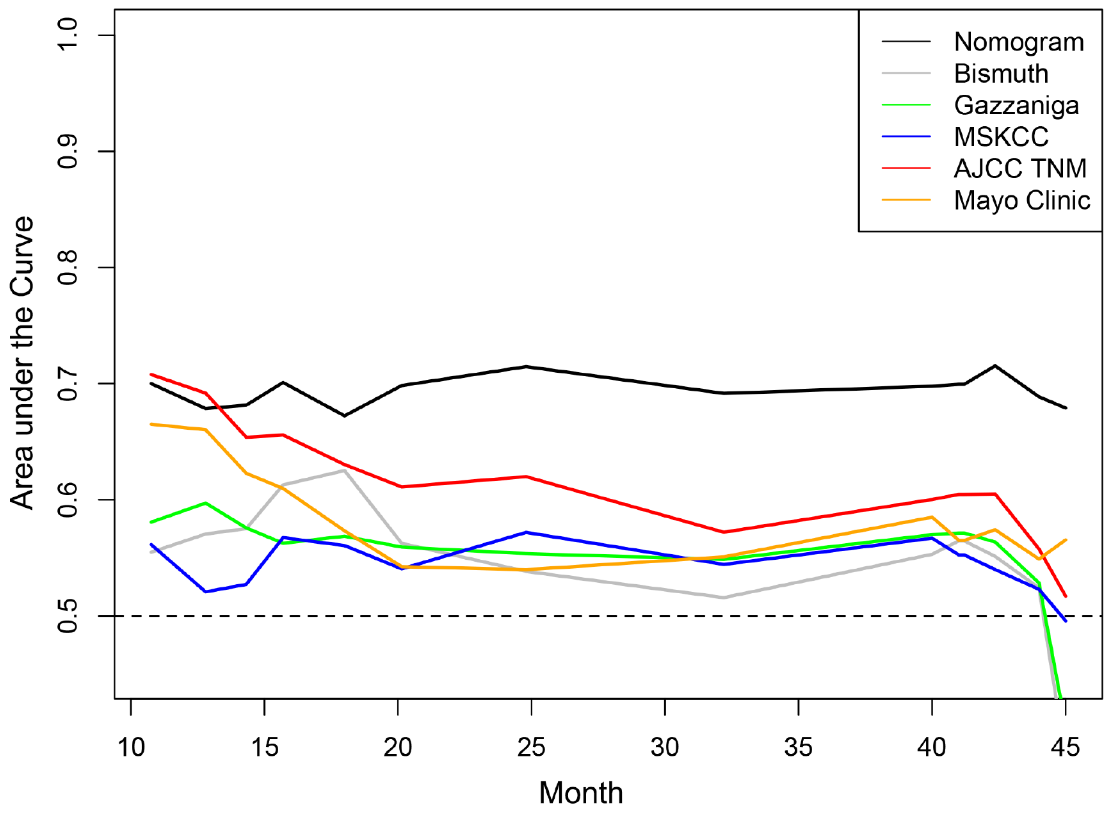

Supplementary Figure S3: Comparison of the time-dependent areas under the ROC curves of prediction by the nomogram and by the conventional staging systems in the internal validation cohort (N = 93).

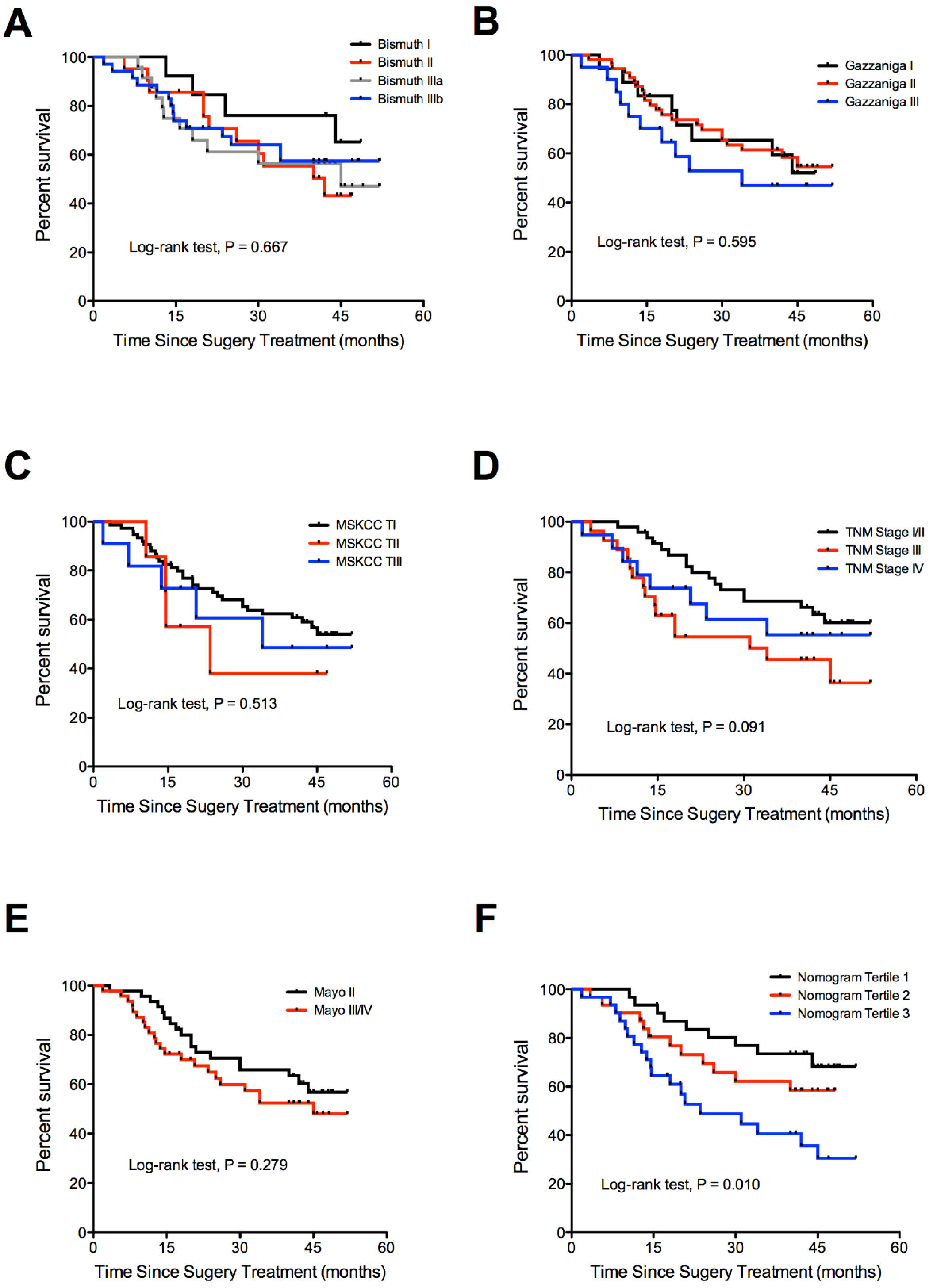

Supplementary Figure S4: Kaplan-Meier survival curves for nomogram and the conventional staging systems in the internal validation cohort. (**A.** Bismuth-Corlette; **B.** Gazzaniga; **C.** MSKCC; **D.** AJCC TNM (seventh edition); **E.** Mayo Clinic; **F.** nomogram).

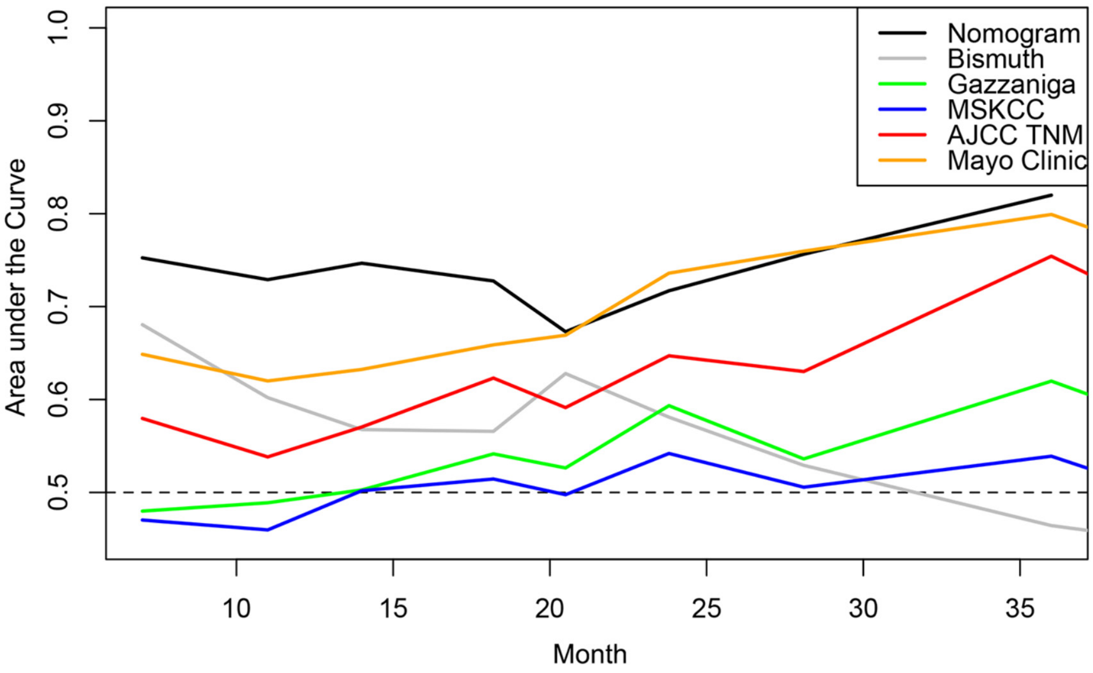

Supplementary Figure S5: Comparison of the time-dependent areas under the ROC curves of prediction by the nomogram and the conventional staging systems in the external validation cohort (N = 84).

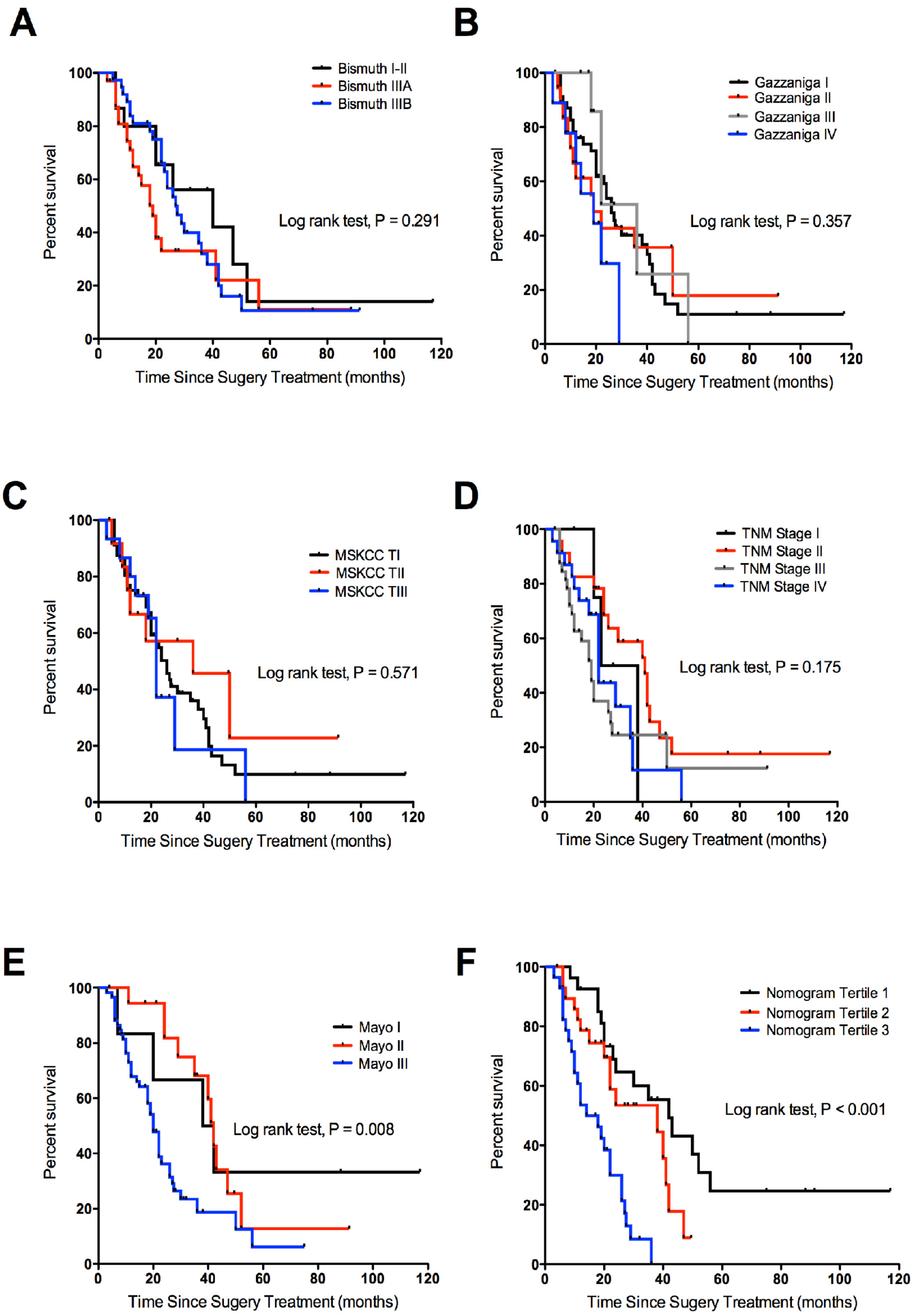

Supplementary Figure S6: Kaplan-Meier survival curves for nomogram and the conventional staging systems in the external validation cohort. (**A.** Bismuth-Corlette; **B.** Gazzaniga; **C.** MSKCC; **D.** AJCC TNM (seventh edition); **E.** Mayo Clinic; **F.** nomogram).
